# Port site herniation of the small bowel following laparoscopy-assisted distal gastrectomy: a case report

**DOI:** 10.1186/1752-1947-2-48

**Published:** 2008-02-14

**Authors:** Tsuyoshi Itoh, Nobuaki Fuji, Hiroki Taniguchi, Taiji Watanabe, Toshiyuki Kosuga, Kingo Kashimoto, Kazuyo Naito

**Affiliations:** 1 Department of Surgery, Kyoto Prefectural Yosanoumi Hospital, Otokoyama Yosano-cho, Yosa-gun, Kyoto 629-2261, Japan

## Abstract

**Introduction:**

Port-site herniation is a rare but potentially dangerous complication after laparoscopic surgery. Closure of port sites, especially those measuring 10 mm or more, has been recommended to avoid such an event.

**Case presentation:**

We herein report the only case of a port site hernia among a series 52 consecutive cases of laparoscopy-assisted distal gastrectomy (LADG) carried out by our unit between July 2002 and March 2007. In this case the small bowel herniated and incarcerated through the port site on day 4 after LADG despite closure of the fascia. Initial manifestations experienced by the patient, possibly due to obstruction, and including mild abdominal pain and nausea, occurred on the third day postoperatively. The definitive diagnosis was made on day 4 based on symptoms related to leakage from the duodenal stump, which was considered to have developed after severe obstruction of the bowel. Re-operation for reduction of the incarcerated bowel and tube duodenostomy with peritoneal drainage were required to manage this complication.

**Conclusion:**

We present this case report and review of literature to discuss further regarding methods of fascial closure after laparoscopic surgery.

## Introduction

Bowel herniation through the fascial defect created by the entry of trocars is now recognized as a rare but potentially serious complication of laparoscopic surgery [1]. Although port site herniation is an infrequent complication, there are still some reports of port site herniation after these procedures, even with closure of trocar sites[1,2]. The following report describes a case of a trocar site hernia that evolved into leakage from the duodenal stump after laparoscopy-assisted distal gastrectomy (LADG). Progression occurred because of complete obstruction of the incarcerated bowel after a Roux-en-Y reconstruction. We describe the significance of complete closure of the fascial defect at the trocar site including the peritoneum in the prevention of this condition, as well as the importance of early diagnosis to avoid serious subsequent events.

## Case presentation

**Figure 1:**
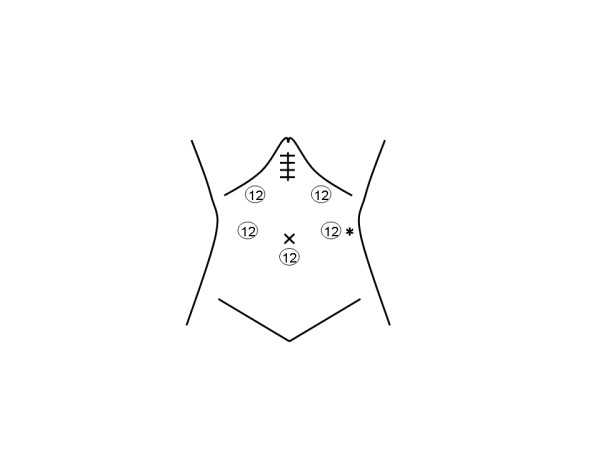
Schematic view of port placement during surgery. Arabic number indicates the size of the port (mm). Herniation occurred at the port site indicated with an asterisk.

An 80-year-old man was found to have early gastric cancer during his yearly check-up by gastrointestinal endoscopy. He was 158 cm in height and weighed 62 kg. Gastrointestinal endoscopy showed a depressed lesion that was diagnosed as early gastric cancer by pathological examination of biopsy specimens. He underwent LADG with regional lymph node dissection (D1 including the nodes surrounding the origin of left gastric artery). A 12-mm trocar for the laparoscope was placed in the umbilicus. Pneumoperitoneum was then established with carbon dioxide and the intraperitoneal pressure was maintained at 10 mm Hg. Two more 12-mm trocars were inserted in the midclavicular line below the costal margin and 2 cm above the umbilicus on each of both flanks and were used for active surgical instruments. All trocars used were the non-bladed type. The specimen was removed through a small medial incision which was 55 mm in length placed after resection of the stomach, and then Roux-en-Y reconstruction (RY) was carried out (Fig. 1). A tubular shaped drainage tube 10 mm in diameter was inserted and placed through the upper trocar site made on the right flank. Wound defect at the umbilical port site was sutured completely including the peritoneum with 0 absorbable suture and fascial incisions at all other trocar insertion sites were closed with 2-0 absorbable sutures. Surgical duration was 263 min, and the volume of blood loss was less than 50 mL with no blood transfusion.

**Figure 2:**
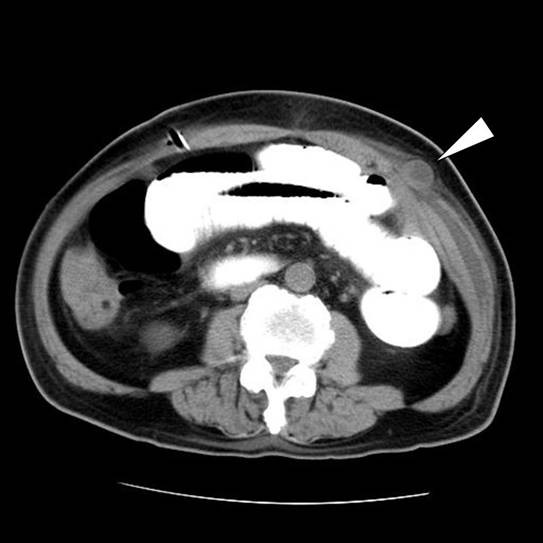
Computed tomography (CT) shows the enlarged duodenum and a mass lesion protruding into the muscular layer of the abdominal wall (arrowhead).

**Figure 3:**
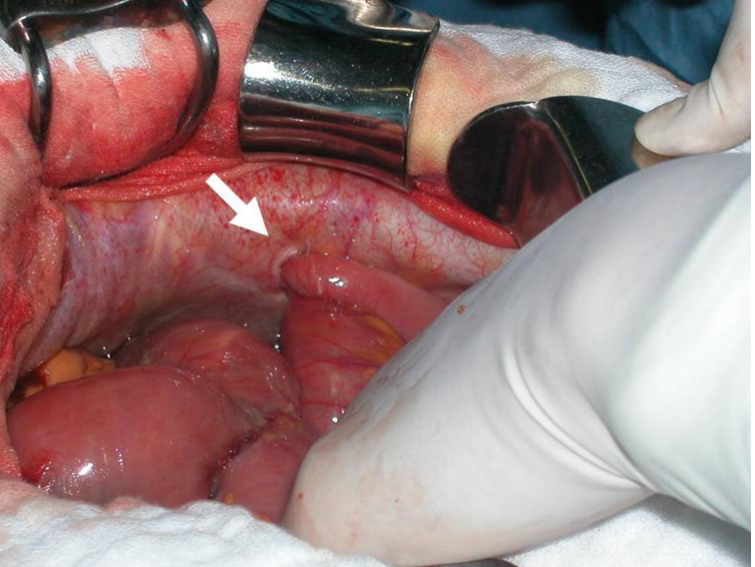
Intraoperative finding showing complete obstruction of the bowel due to incarceration into the peritoneal defect at the trocar site (arrow).

Postoperatively, the patient complained of an acidic feeling in his stomach; however, there were no remarkable abnormalities on biochemical examination of serum. Radiological findings did not suggest bowel obstruction until 3 days postoperatively, although mild symptoms such as general malaise and vague abdominal pain were reported on day three. However, on day 4, the patient started to complain of upper abdominal pain and developed a high grade fever (38°C). Complete obstruction of the small bowel and leakage of contrast media were demonstrated by Gastrografin swallow and subsequent abdominal computed tomography (CT). CT also showed a mass lesion at the trocar insertion site on the upper left flank, suggesting herniation through the port site (Fig. 2). Marked dilatation of the duodenum including the horizontal part and second portion was observed. A diagnosis of staple failure of the stump of the duodenum and port-site herniation of the small bowel was made, and exploratory laparotomy was carried out. A small medial incision that had been made at the initial surgery was extended downward to the umbilicus to open the peritoneal cavity. As we expected, the small bowel was incarcerated into the peritoneal defect in the abdominal wall created by the trocar placed in the left upper flank leading to complete obstruction of the bowel (Fig. 3). Part of the jejunum 30 cm distal from the ligament of Treitz herniated around the fascial stitch, which still existed at the time of the re-exploration. The peritoneal cavity was contaminated with intestinal juice. Close examination after reduction of the incarcerated bowel did not demonstrate necrosis of the intestine, and thus, we decided not to resect this lesion. Leakage of intestinal juice through a pinhole fistula at the duodenal stump was also observed. Tube duodenostomy was performed with an omental patch used for closure of the fistula. The peritoneal defect was also closed. The postoperative course was fairly good without high output of the intestinal juice leakage or sepsis. The patient remained in the intensive care unit for 5 days after re-operation, and was then transferred to the general ward.

## Discussion

Port-site herniation, which is one of the major complications after laparoscopic procedures [1], sometimes develops into serious complications, such as bowel obstruction due to incarceration into the fascial defect at the port site. Boughey et al. have reported four cases of Richter's hernia that occurred at a port site after laparoscopic surgery [1]. They reviewed previous reports and found the incidence to be 0.2 to 3%. A report describes the incidence of hernia as 0.23% for 10-mm trocar use, rising to 3.1% for the 12-mm trocar [2] suggesting that the wound created by a larger port carries a greater risk of herniation. Most surgeons now routinely close the fascia of port sites to prevent this complication [2]. According to previous reports, port site herniation apparently happens more often with the use of bladed type trocars than non-bladed type trocars [3]. Indeed, Kolata demonstrated that the wounds made by the non-bladed trocar were narrower than those created by cutting tip trocars in a pig experimental model [4]. Several reports even concluded that port sites created by non-bladed trocars do not require fascial closure [3]. However, the current case suggests that thick preperitoneum is a potential space that allows for the development of bowel herniation even with the use of non-bladed type trocars. A previous report also described port-site herniation, despite the closure of the superficial layer of the fascial defect [5]. The current case did not demonstrate any of the risk factors suggested previously [6]; 1) enlargement of a port site to remove specimen; 2) glucose intolerance; 3) obesity; or 4) extensive manipulation of the trocar during relatively prolonged surgical duration, which might have enlarged the trocar site and thus induced bowel herniation. Therefore, we recommend closing the fascial defect, including the peritoneum, especially if the trocar size is more than 10-mm and in the presence of any of the risk factors described above. However, it is sometimes difficult to completely close the defect, including the peritoneum, especially in obese patients. Shaher reviewed different wound closure techniques by a literature search [7]. In this review, old methods using classical instruments including Deschamps needle are also useful as well as special wound devices designed for port site closure. Elashry et al. described a prospective randomized study demonstrating that the Carter-Thomason device was faster and resulted in fewer port-closure-related complications among eight different techniques tested [8]. Insertion of a SURGICEL plug into the muscular layer of trocar wounds has also been proposed by Chiu et al [9]. Alternatively, tangential insertion of a trocar through the abdominal wall might be effective in reducing the size of fascial defects. Moreover, recent publications have demonstrated that radially expanding type trocars could be useful to avoid the necessity of closing the fascial defect [10].

Symptoms of trocar-site herniation vary depending on the severity of bowel obstruction. Mild symptoms such as slight nausea and vague abdominal pain, both of which are most frequently seen in the early normal postoperative course after abdominal surgery, could be the first and only complaints at the early stage of this complication. Thus, the diagnosis may be delayed. In our case, mild abdominal pain with general malaise might have been symptoms related to the early stage of the onset. Abdominal CT showing the enlarged duodenum also suggested that leakage from the duodenal stump occurred due to the obstruction of the distal bowel. Thus, severe complication might have been avoided, if early diagnosis had been made. Although the benefit of Roux-en-Y is apparent [11], the duodenal stump could be vulnerable to leakage due to increased intrabowel pressure. Therefore, careful management of the postoperative course is warranted, especially after procedures involving division of the bowel such as LADG. Moreover, special attention should be paid in patients with risk factors for port site hernia such as obesity, aggressive manipulation through the port sites, and prolonged surgery.

## Conclusion

Port-site herniation is a potentially dangerous complication after laparoscopic procedures. Careful management of the postoperative course is recommended especially for patients with risk factors such as obesity and extensive manipulation of the trocar during prolonged surgical duration.

## Competing interests

The author(s) declare that they have no competing interests.

## Authors' contributions

TI, NF, HT and TK performed the first and second operation. TI and KK were responsible for the postoperative management. TI, HT, TW, and KN were involved in editing the manuscript. All authors read and approved the final manuscript.

## Consent

Written informed consent was obtained from the patient for publication of this case report and any accompanying images. A copy of the written consent is available for review by the Editor-in-Chief of this journal.
